# On Transfered Irritation

**Published:** 1819-08

**Authors:** 


THE LONDON
### Medical and Physical Journal.

2 OF VOL. XLII.] AUGUST, 1819. [NO. 246.

"For many fortunate discoveries in medicine, and for the detection of numerous "errors, the world is indebted to the rapid circulation of Monthly Journals; "and there never existed any work to which the Faculty in EUROPE and "AMERICA were under deeper obligations than to the Medical and Physical "Journal of London, now forming a long, but an invaluable, series." RUSH.

FOR THE LONDON MEDICAL AND PHYSICAL JOURNAL.

### *On Transfered Irritation.* By DR. KINGLAKE.

MEDICAL practitioners of the earliest period have noticed the transferable property of local irritation from one part of the animal frame to another. It seems to be a law of the animal economy, that morbid excitement relieves itself either by exhaustion, by diffusion, or by acting with predominant violence on a part more or less remote from that which may be primarily affected. The most salutary and direct termination, is by exhausting the degree of vital power on which the morbid action depends. When this takes place, a spontaneous or natural cure occurs; and, happily for mankind, by far the largest portion of disease that arises, ends in this mild and desirable manner. When the diseased action lingers and acquires an habitual continuance, or when by its violence it disorganizes in any measure the healthy structure of the part in which it presents, it becomes too untractable to give way to the salutary influence of exhaustion, but remains the effect of an accustomed or vitiated action.

The diseased state, in whatever circumstances it may occur, is a degeneracy from the natural condition of life; and it is at all times desirable that it should terminate with all possible speed. To neglect it, may be hazardous; but to protract it, is to incur an injury, the extent of which can neither be foreseen nor defined. It is the province of the healing art, to remove obstacles to either natural or artificial modes of cure, and to furnish every facility for accomplishing the salutary object. Cases are often so circumstanced as to require seasonable assistance; and, if it be unduly delayed, the opportunity for benefiting may be either lost or rendered unavailing by such omission. It should be remembered, that, when a disease has once arisen, it becomes of itself a stimulus for its own continuance, if it be not speedily dissipated, either by exhaustion, diffusion, or transference. If suffered to remain, it will be so located and established in the situation where it originated, as to be at least durable, if not positively incurable.

The cause of disease must, in the first instance, be sufficiently powerful to supersede or overcome the healthy state; but it is not necessary that the morbid cause should be incessantly applied to sustain the effect. The diseased state itself will be fully sufficient to carry on its own action, until it be either spontaneously worn out, or counteracted and subdued by adequate remedies. Febrile affections, derivable from the usual sources of epidemic, endemic, or sporadic diseases, are independent of their respective causes, whether from marsh miasma, aerial impurity, or personal contagion. After the fever has once occurred, neither the continued nor renewed application of the cause has any additional effect. The progress of the disease is the same, neither increasing nor diminishing in violence by the presence or absence of the morbid cause by which the disease was induced.

Gouty or inflammatory excitement of the ligaments and tendons is at present almost the only disease that is regarded as salutary, and which has obtained, in some measure, both a medical and popular sanction for its existence, its encouragement, and indefinite duration. The exanthematous affections have likewise somewhat shared in the leaven of remedial virtue. It is difficult to imagine how it could be supposed that an afflicting form of disease could ever prove beneficial; but, if the solecism be admitted, it is perhaps less extraordinary than the notion that one disease should vicariously perform the services of another. It is yet to be satisfactorily explained, how it is possible that disease can improve the healthy state! To say that previous disease existed, and that inflamed ligaments and tendons, or eruptions on the surface of the skin, removed that disease, is no more than stating an extension of morbid action to those several parts. But, does not this position take for granted what is not proved, and which can neither be logically insisted on, nor rationally conceded? Both arthritic and cutaneous excitement are frequently occurring without any previous sense of ailment; and, if the degree of violence by which they may be attended should disorder the general frame, or occasion on any particular part of the system a stronger impression than on another, it must be considered as the effect, as the diffusion of these local affections, and not as an integrant part of the original disease itself. Local disease is one thing, its dissemination is another; and it should be the leading object of medical endeavour to restrain and extinguish it at the place of its origin or birth.

Phrenitis, peripneumonia, carditis, gastritis, &c. should not be suffered to extend the morbid excitement by which they are respectively characterized, to the system at large. It should, when possible, be instantly subdued, and not be allowed to augment and diffuse itself over the general frame. Disease strengthens with its growth, "*vires acquirit eundo*;" and, if permitted to proceed, will ultimately become much more formidable than would have been the case, if seasonably repressed and subdued.

The extinction of gouty and pustular inflammation, before the heart and arteries may be excited by its stimulating influence, and consequently before the visceral functions be disordered, is surely preferable to having to cope with all these diseased effects. If remedies were promptly efficient, diseases would be advantageously crushed in their bud; time would not be afforded for unfolding their full form and violence. "*Venienti occurrite morbo*," should be the assiduous attempt; and, whenever practicable, should not be omitted. Prevention is better than cure; and the means for effecting the former are much less fallible than those which offer for accomplishing the latter. Morbid irritations are various in form and afflicting in severity; but the sensations of pain in a distempered state of nervous power, are almost infinite, and not less distressing. When the vital action of the nervous system is so disordered, in respect either of due energy or regular distribution of sensorial power, every variety of feeling may be experienced; and, in the endless modification of them, they may be selected and grouped in such a manner, as would characterize any description of complaint that could be either imagined or stated. It is highly probable that, in this exquisitely sentient state of the nervous system, even the actions of life themselves are felt, and that they are really attended with acute suffering. It is not unusual to hear patients, thus overwhelmed with morbid feeling, complain of darting, creeping, drawing, twisting, rending, motions in various parts of the frame, passing and re-passing with electric celerity from one situation to another. If, in such circumstances, either the muscular, the ligamentous, the tendinous, or membranous, structure should, from being more particularly excited by the stimulus of diseased sensation, become the seat of either preternatural irritation or of inflammatory action, corresponding disease will be produced, with which the heart and arteries may so sympathise as to induce general affection.

Much expression of that sort of pain which arises from a morbidly sentient state of the nervous system, is often made without its reality being at all evinced by an increased action of the arterial system; and, as long as this is the case, the animal functions remain for the most part undisturbed: digestion and nutrition proceed, and no immediate danger of life, in circumstances otherwise as deplorable as generally irremediable, can be apprehended. It is in this distressing state of distempered sensibility, (of which the quick recession of the sensitive-plant on the slightest touch is but a faint emblem,) that much imaginary complaint is made, and stated with a precision that would become the accuracy of mathematical demonstration. These actual pains and feeling descriptions often lay the ground of fallacious speculations, in estimating the true nature of diseases. Arthritic inflammation, occurring in such circumstances of diseased sensation, will, in the imagination of the patient, promiscuously dart, with explosive rapidity, piercing pain to the stomach, brain, heart, or some other vital organ, to the great terror of both patient and spectator. A real connexion, a physiological sympathy, is supposed to subsist between the gouty joint and the pained viscus, and a destructive transference of disease to the vital organ is dreaded! The utmost earnestness is shown in endeavouring to station the menacing enemy on the affected joint, and thus to ward off the deadly blow that might otherwise reach the unhappy patient. The pustular inflammation induced by rubbing on the region of the chest tartarized antimony, formed into an ointment with hog's-lard, recently excited in the sensations of a patient whose nervous system was agonized by excess of feeling, a severity of pain, that was described as resembling that which might be occasioned by driving a long nail through the pectoral viscera! Some degree of pain might be experienced; but, independently of the shattered and disordered state of the nervous system, it was what would not have been noticed by the patient; nor, in point of fact, does a similar application induce scarcely any complaint in the most delicate children.

To regard pain, abstractedly considered, as a legitimate datum on which to argue the fugitive and fluctuating nature of disease, is an unwarrantable assumption. It is much too vague and equivocal in its nature, cause, origin, and extension, to be admitted as a just ground on which to determine the true character and effects of erratic pains, however proteiform and afflicting may be the suffering by which they may be accompanied. These variable and excursive pains almost exclusively belong to a disturbed nervous system, and ought never to be adduced in proof of any hypothesis respecting the laws and conditions by which the transference of disease is regulated. The theories must indeed be wildly visionary, in which casual, transient, and evanescent pains, occurring in a state of distempered sensibility, should be made the basis of positive disease. Like *igniis fatui*, they will betray the deluded enquirer into an inextricable labyrinth of misapprehension and error.

Two instances have lately occurred in my practice, of the transfering and counteracting influence of inflammatory action taking place in a situation remote from the part that was first affected. The medical practitioner and the physiologist must feel an interest in obtaining additional proof of the possibility of one inflammatory ailment having the effect of gradually diminishing, and at length of wholly superseding, another. The cases referred to, are those of females after parturition. In both instances, as if nature were proceeding by an established law from which there could be no deviation, high inflammatory excitement arose in the uterine system on about the fourth day after the accouchement, accompanied with a tumid abdomen, scanty lactition; and, in one of the cases, with an inability to pass water, requiring its occasional removal by the catheter. In these circumstances, much general disease prevailed: indeed, the most unpromising aspect of puerperal fever was exhibited. Repeated bleedings, a soluble state of the bowels effected by castor-oil and clysters, and blistering the parietes of the abdomen, were the means employed for obtaining relief. Much apparent danger of life, however, attended both these cases, until an inflammatory tumor discovered itself on one of the arms. The whole of the upper-arm, from the shoulder-joint to the elbow, was soon occupied by the tumefaction. It was not acutely painful. The affected limb, in both cases, soon became enormously swollen, losing all power of voluntary motion, and rapidly proceeded to suppuration. A considerable quantity of matter formed, which was vented, and the wound resulting from the abscess continued to yield a copious discharge for several weeks. Every symptom of puerperal disease gradually diminished, and finally subsided, after the phlegmonous tumor appeared on the arm. The relief afforded by the transfer was all that could be desired; and in both instances, after the lapse of a few weeks, it terminated in the complete recovery of the patient.

These cases furnish strong proof of the influence which inflammatory action in one situation of the body is capable of exerting over another. They seem to evince, that local preponderance on a fresh part is necessary to suspend and remove an inflammatory action that might have previously existed. General diffusion, in the form of febrile excitement, does not overcome the concentrated violence at the part originally affected; but, if the excitement should amass in a particular situation at a remote distance from the seat of the primary disease, it appears, by the agency of derivation or revulsion, to have the effect of exonerating the disordered part from the heavy pressure of disease, under which, without such critical and salutary relief, life itself might be endangered. In these cases, inordinate uterine excitement produced the puerperal disease that occurred; and, if that state had continued unrelieved, the most imminent risk of its becoming destructive would have been incurred. The sympathetic influence of the uterus over the general system is, perhaps, superior to what is exerted by any other viscus; it is therefore, amidst the extensive liability for taking a sympathetic disease, uncertain on what particular part it may fall. The extremities are probably the most remedial situations for such transference of disease. The lower extremities occasionally sympathize with uterine irritation so as to become painfully œdematous; of which the phlegmasia dolens is an example: but it has not been so common for the upper limbs to be the seat of metastasis in uterine disease. The natural tendency, however, to such a curative deposit on the extremities on these occasions, suggests a practical hint as to the propriety of inviting it thither, by blistering, or by any other mode of exciting inflammatory action, instead of instituting, as usual, counter-irritation over the region of the abdomen. It has been a long time my practice, and apparently with good effect, to apply, in visceral diseases, blisters to the inside of the lower extremities. It may also be advisable to endeavour to aid, by making similar applications to the upper limbs. It appears reasonable to expect, that more benefit would result, generally speaking, not only in uterine, but in all other visceral affections, by remote counter-excitement, than from the prevailing practice of employing it in the vicinity of the diseased organ. Thus, it would probably prove more remedial to blister the extremities in all visceral inflammations, and to apply cold to the external regions of these parts. This practice is certainly most salutary in phrenitis and in enteritis; and, perhaps, it may be safely extended to acute affections of the chest. The most unpromising case of peritonitis that ever fell under my observation, was speedily and effectually relieved by the free topical application, over the region of the abdomen, of cloths wetted in cold vinegar and water.

Much still remains to be learnt as to the best and most successful mode of treating inflammatory disease, whether stationed in certain parts or sympathetically transfered to others. It is obviously proper, under all circumstances, to aim at the earliest possible cure of all inflammatory as well as other affections. Their continuance is fraught with mischief. Their original attack is injurious; and, by delay, incurable ailment may be produced, either in the part first affected, or by the diseased sympathies which are apt to be engendered in other portions of the system. The diseased state is not easily generated. The *vis insita* of life and health tenaciously keeps its ground; and when it is positively disordered, every practicable assistance should be afforded for its restoration and establishment. The devious course should be retraced, until the healthful state shall regain its natural ascendancy and fixation.

*Taunton; June 19th,* 1819.

